# Lupin Peptide T9 (GQEQSHQDEGVIVR) Modulates the Mutant PCSK9^D374Y^ Pathway: in vitro Characterization of its Dual Hypocholesterolemic Behavior

**DOI:** 10.3390/nu11071665

**Published:** 2019-07-20

**Authors:** Carmen Lammi, Carlotta Bollati, Davide Lecca, Maria Pia Abbracchio, Anna Arnoldi

**Affiliations:** 1 Department of Pharmaceutical Sciences, University of Milan, 20133 Milan, Italy; 2 Department of Pharmacological and Biomolecular Sciences, University of Milan, 20133 Milan, Italy

**Keywords:** bioactive peptides, HepG2, LDLR, lupin, PCSK9

## Abstract

GQEQSHQDEGVIVR (T9) is a peptide originated by the tryptic digestion of lupin β-conglutin that is absorbed in human intestinal Caco-2 cells. A previous study has shown that T9 impairs the protein–protein interaction between mutant D374Y Proprotein Convertase Subtilisin/Kexin 9 (PCSK9^D374Y^) and the low-density lipoprotein receptor (LDLR), thus exerting a hypocholesterolemic effect. Moreover, a bioinformatic study predicting that T9 may potentially act as an inhibitor of 3-hydroxy-3-methylglutaryl CoA reductase (HMGCoAR), has suggested a complementary cholesterol-lowering activity. The present study demonstrates that T9 inhibits in vitro the HMGCoAR functionality with an IC_50_ value of 99.5 ± 0.56 µM. Through the inhibition of either HMGCoAR or PCSK9^D374Y^ activities, T9 enhances the LDLR protein levels leading to an improved ability of HepG2 cells transfected with the mutant PCSK9^D374Y^-FLAG plasmid to uptake extracellular LDL with a final cholesterol-lowering effect. In addition, T9 modulates the PCSK9^D374Y^ signaling pathway in transfected HepG2 cells leading to a decrease of PCSK9^D374Y^ and HNF-1α protein levels. All these results indicate that the hypocholesterolemic effects of T9 are due to a dual mechanism of action involving either the modulation of the PCSK9^D374Y^ or LDLR pathways. This may represent an added value from a therapeutic point of view.

## 1. Introduction

Reducing low-density lipoprotein cholesterol (LDL-C) is a priority in managing patients with severe hypercholesterolemia and at high risk of cardiovascular disease and disability [1]. The traditional approach consists of inhibiting the activity of 3-hydroxy-3-methylglutaryl CoA reductase (HMGCoAR), which is the rate-limiting enzyme of the cellular cholesterol biosynthetic pathway [2]. This approach represents an efficient way to reduce serum cholesterol levels through the increased ability of the LDL receptor (LDLR) to uptake extracellular LDL [3]. In this context, literature provides some examples of natural compounds targeting the HMGCoAR activity, such as lupin, soybean, and amaranth peptides, or monacolin K in red yeast rice extract [4,5,6,7].

In 2003, however, another promising protein target, i.e., proprotein convertase subtilisin/kexin type 9 (PCSK9), had appeared on the scene and soon the correlation between its activity and circulating LDL-C levels had been clarified [8]. The main role of PCSK9 is the degradation of LDLR protein with a subsequent increase of the LDL-C levels [9,10,11].

It is important to observe that some natural PCSK9 mutations affect its affinity for the LDLR. In particular, the gain of function (GOF) mutant D374Y binds more avidly to the LDLR than the wild-type (WT) PCSK9. The consequence is an approximately tenfold increased capacity of reducing the LDLR protein level [9]. Accordingly, subjects with this PCSK9^D374Y^ mutation have very high LDL-C levels and are at high risk of atherosclerosis. In light of these observations, PCSK9 is currently considered a new target for the treatment of hypercholesterolemia, and the development of novel PCSK9 inhibitors is certainly a challenging goal [12]. In this context, lupin peptides and berberine have been demonstrated to modulate the PCSK9 pathway with different mechanisms [13,14].

In this panorama, lupin protein hydrolysates, obtained by treating the proteins with pepsin and trypsin, show synergistic hypocholesterolemic effects through the modulation of both HMGCoAR and PCSK9 targets [4,13,15,16]. In fact, either the peptic or tryptic hydrolysates drop the HMGCoAR activity in vitro, inducing the LDLR pathway, reducing the PCSK9 pathway at the intracellular level, and improving the ability of hepatic HepG2 cells to uptake the LDL from the extracellular environment [4,16]. Furthermore, these lupin hydrolysates impair the protein–protein interaction (PPI) between PCSK9 and LDLR [15]. Although it seems feasible that these complementary activities might be due to the synergistic effects of different peptides in the hydrolysates, it cannot be excluded that some single peptides endowed of a dual inhibitory behavior may be present inside these hydrolysates, which have a very complex composition.

GQEQSHQDEGVIVR (T9) is a peptide, deriving from the hydrolysis of β-conglutin with trypsin, showing a pI of 4.3, a hydrophobicity value of 25.97 Kcal mol^−1^, and a net charge of −2 at pH 7.0. Its 14-aminoacids sequence comprises a polar negatively charged N-terminal, containing a glutamate residue, and a nonpolar positively charged C-terminal, containing an arginine residue. Suitable experiments have shown that T9 is absorbed by enterocytes and results to be stable to the metabolic degradation operated by intestinal peptidases [13]. Two previous in silico studies have predicted the potential ability of T9 to inhibit two key targets in hypercholesterolemia treatment, such as PCSK9 and HMGCoAR [13,15]. Based on these considerations, in a previous paper T9 had been synthesized and demonstrated to impair the PPI between PCSK9 or PCSK9^D374Y^ and the LDLR [17]. In light of these observations, this work was aimed at a deeper elucidation of the dual inhibitory effects of this interesting peptide. In detail, the first goal of the work was the assessment of the capacity of T9 to modulate the HMGCoAR activity and the evaluation of the effects on the LDLR-pathway using HepG2 cells. Moreover, since T9 is able to impair the PPI between PCSK9^D374Y^ and the LDLR, a second objective was to investigate the effects of T9 on the modulation of the PCSK9^D374Y^ pathway after transfection of pcDNA3+PCSK9^D374Y^-FLAG plasmid into HepG2 cells, in order to produce the mutant PCSK9 in the in vitro liver system.

## 2. Materials and Methods

### 2.1. Materials

See Supplementary Materials for further details on materials and methods.

### 2.2. HMGCoAR Activity Assay

The assay buffer, NADPH, substrate solution, and HMGCoAR were provided in the HMGCoAR Assay Kit (Sigma). The experiments were carried out as previously described [4]. See Supplementary Materials for further details.

### 2.3. Cell Culture Conditions and Transfection

The HepG2 cell line was bought from ATCC (HB-8065, ATCC from LGC Standards, Milan, Italy) and was cultured following the conditions previously described [4]. Detailed information is reported in the Supplementary Materials. A total of 3 × 10^4^ HepG2 cells/well were seeded in 96-well plates. The following day, cells at a 70–90% confluence were transfected with the mixture containing 1.0 µg pcDNA3+PCSK9^D374Y^-FLAG plasmid and 2.0 μL TurboFect Transfection Reagent in 100 µL of serum-free DMEM for 48 h. After 24 h, transfected HepG2 cells were treated with peptide T9 (100 µM) and incubated for 24 h at 37 °C under 5% CO_2_ atmosphere.

### 2.4. Western Blot Analysis

Transfected HepG2 cells (1.5 x 10^5^ cells/well) were treated with 100 μM T9 for 24 h. After each treatment, cells were assessed in western blot experiments as previously described [4]. See Supplementary Materials for further details.

### 2.5. In Cell-Western

Transfected HepG2 cells were treated with 100 µM T9 or vehicle (H_2_O) for 2 h at 37 °C under 5% CO_2_ atmosphere. Treated HepG2 cells were then fixed in 4% paraformaldehyde for 20 min at room temperature (RT) and assessed for in-cell western experiments as previously described [5]. See Supplementary Materials for further details.

### 2.6. Assay for the Evaluation of Fluorescent LDL Uptake by HepG2 Cells

After transfection, a total of 3 × 10^4^ HepG2 cells/well were seeded in 96-well and treated with 100 μM T9 or vehicle (H_2_O) for 24 h. LDL-Uptake Assay were carried out as previously described [4]. See Supplementary Materials for further details.

### 2.7. Statistical Analysis

Statistical analyses were carried out by One-way ANOVA (Graphpad Prism 6), followed by Dunnett’s test. Values were expressed as means ± SD, *p*-values < 0.05 were considered to be significant.

## 3. Results

### 3.1. T9 Drops the HMGCOAR Activity In Vitro

**Figure 1:**
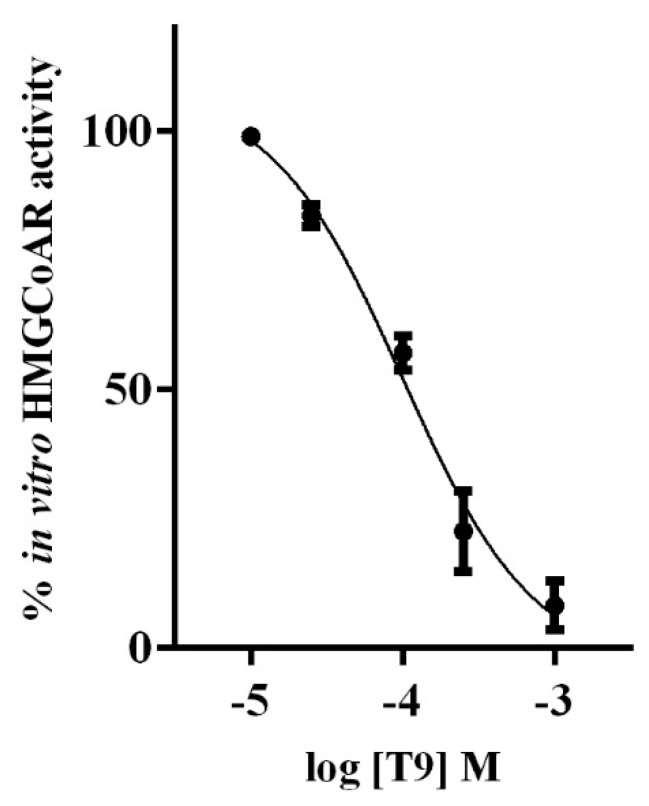
In vitro inhibitory effect of T9 on HMGCoAR activity.

In vitro experiments were performed in order to confirm the in silico study according to which T9 was a potential inhibitor of the HMGCoAR activity [13]. Peptide concentrations ranging from 10^−5^ to 10^−3^ M were tested. Figure 1 indicates that T9 reduced the HMGCoAR activity in a dose-dependent manner and with an IC_50_ value equal to 99.5 ± 0.56 µM.

### 3.2. Peptide T9 Modulates the PCSK9^D374Y^ Protein Level on HepG2 Cells

**Figure 2:**
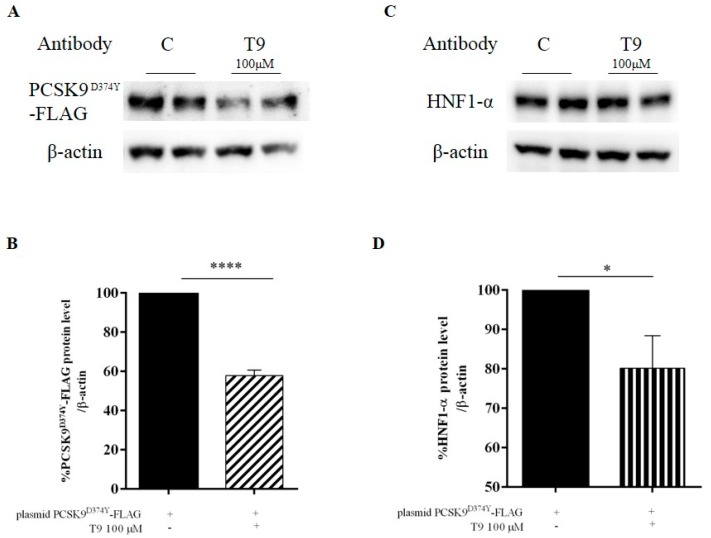
Effects of T9 on PCSK9^D374Y^-FLAG (**A,B**) and HNF-1α (**C,D**) protein levels. Transfected HepG2 cells were incubated with T9 for 24 h. PCSK9^D374Y^-FLAG, HNF-1α, and β-actin were detected using specific anti-FLAG, anti-HNF-1α, and anti-β-actin primary antibodies, respectively. Each protein signal was quantified and normalized with β-actin signals. The data points represent the averages ± S.D. of four independent experiments in duplicate. (*) *p* < 0.05, (****) *p* < 0.0001 vs. untreated transfected samples (C: control cells).

Experiments were performed for assessing the capacity of peptide T9 to modulate the PCSK9^D374Y^ pathway in transfected (pcDNA3+PCSK9^D374Y^-FLAG plasmid) HepG2 cells. Mature PCSK9^D374Y^-FLAG was detected by immunoblotting using a specific primary antibody against the FLAG tag. As Figure 2A,B shows, the treatment with T9 (100 μM) reduced the PCSK9^D374Y^-FLAG protein level by 41.2 ± 2.6% compared to the untreated transfected HepG2 sample. In parallel, T9 decreased the HNF-1α level by 19.8 ± 8.2% at 100 μM (Figure 2C,D).

### 3.3. T9 Modulates the LDLR Pathway on HepG2 Cells Transfected with PCSK9^D374Y^

**Figure 3:**
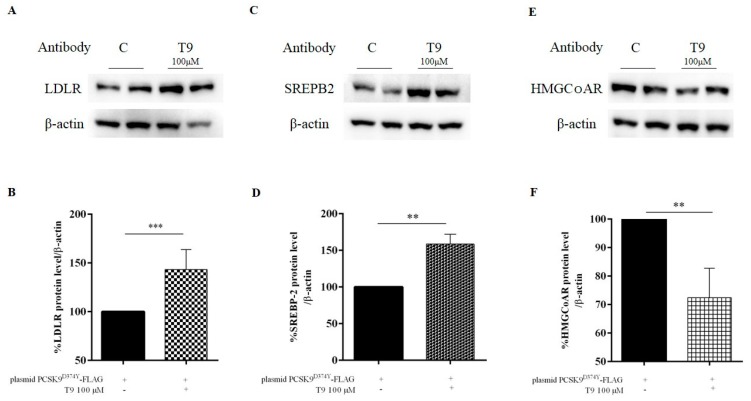
Effects of T9 on LDLR (**A,B**), SREBP-2 (**C,D**), and HMGCoAR (**E,F**) protein levels. Transfected HepG2 cells were incubated with T9 for 24 h. LDLR, SREBP-2, HMGCoAR, and β-actin were detected by immunoblotting using anti-LDLR, anti-SREBP-2, anti-HMGCoAR, and anti-β-actin primary antibodies, respectively. Bars represent averages ± S.D. of four independent experiments (two duplicates per sample). (**) *p* < 0.001, (***) *p* < 0.0001 vs. untreated transfected samples (C: control cells).

HepG2 cells were transfected with PCSK9^D374Y^-FLAG-plasmid, and after 24 h, transfected cells were incubated with peptide T9 (100 μM). After 24 h, immunoblotting experiments were performed for evaluating the effects of the treatment on the modulation of the LDLR pathway. Figure 3A,B shows that T9 induced an increase of LDLR protein level by 43.4 ± 2.4% compared to untreated transfected cells and an up-regulation by 46.9 ± 2.6% of the protein level of the N-terminal fragment of SREBP-2 (Figure 3C,D). On the contrary, the same treatment induced a 21.7± 1.1% reduction of the HMGCoAR protein level reduction *vs* untreated transfected cell (Figure 3E,F).

### 3.4. T9 Increases the Ability of Human Hepatic Cells Transfected with PCSK9^D374Y^ to Uptake Extracellular LDL

**Figure 4:**
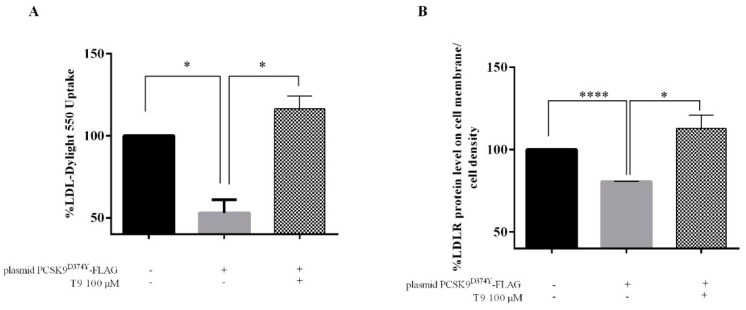
(**A**) Fluorescent LDL-uptake assay after treatment of HepG2 with T9. HepG2 cells were transfected and subsequently incubated with T9 for 24 h. Then, the specific fluorescent LDL uptake was measured by the Synergy H1 fluorescent plate reader. Data points represent averages ± S.D. of three independent experiments in triplicate; (*) *p* < 0.05. (**B**) Cell surface LDLR levels in HepG2 cells. An ICW is a colorimetric cell-based assay where cell membranes are not permeabilized, useful to detect the up-regulation of specific LDLR protein target on the cell surface. Bars represent averages ± S.D. of three independent experiments. (*) *p* < 0.05 and (****) *p* < 0.00001.

The variation of the functional capability of transfected HepG2 cells to absorb LDL from the extracellular environment after the treatments with T9 was investigated by performing a fluorescent-LDL uptake assay. More in details, whereas transfected HepG2 cells showed a 47.0 ± 4.5% reduced capability of uptaking fluorescent LDL compared to untransfected cells (Figure 4A), the treatment with T9 at the concentration of 100 µM completely restored the LDL uptake increasing by 16.4% the ability of transfected cells to absorb LDL compared to the untransfected cells.

This result agrees with the improved amount of active LDLR protein localized on the cell surface of transfected HepG2 cells that were detected by an in-cell western (ICW) assay. Briefly, after 24 h of transfection, a decrease of the LDLR protein level on the cell surface by 19.5% was observed compared to untransfected cells, but the treatment with T9 (100 μM) significantly restored the active LDLR protein level (Figure 3B).

## 4. Discussion

Recently, we have demonstrated that P5 (LILPKHSDAD) and T9 are the first food-derived peptides able to affect the PPI between PCSK9 and the LDLR, determining an increased ability of HepG2 cells to clear extracellular LDL [15]. Surprisingly, T9 is also able to impair the PPI between PCSK9^D374Y^ and the LDLR. In parallel, we have also predicted by in silico experiments that T9 had the potential capability of inhibiting the HMGCoAR activity. All these results have suggested a deeper investigation on the possible multi-target hypocholesterolemic activity of T9 that was assessed by a combination of molecular and functional techniques.

In particular, our results demonstrate that indeed T9 inhibits the HMGCoAR activity, confirming the in silico prediction. In fact, T9 drops the enzyme activity with an IC_50_ value equal to 99.5 ± 0.56 µM (Figure 1). Three other absorbable peptides deriving from the hydrolysis of lupin β-conglutin hydrolysis with pepsin have similar inhibitory activities on this enzyme. Peptides P5, P7 (LTFPGSAED), and P3 (YDFYPSSTKDQQS) reduce in vitro the HMGCoAR activity with IC_50_ values of 147.2, 68.4, and 70 μM, respectively [16,18]. On the other hand, some peptides deriving from soybeans are less active, since IAVPTGVA, IAVPGEVA, and LPYP (from glycinin hydrolysis) inhibit the HMGCoAR activity with IC_50_ values equal to 247, 222, and 300 μM, respectively, whereas YVVNPDNDEN and YVVNPDNNEN (from β-conglycinin) reduce the enzyme activity with IC_50_ values equal to 1500 and 200 µM, respectively [19,20,21]. Also, GGV, IVG, and VGVL, from amaranth protein hydrolysis, exert a potential hypocholesterolemic effect through direct inhibition of the reductase activity. VGVL, the most active, inhibits the HMGCoAR by 50% at 51.9 μM [6].

The ability of T9 to reduce the HMGCoAR activity, together with its ability to affect the PCSK9^D374Y^–LDLR binding in vitro, prompted us to investigate the mechanism of action through which T9 modulates the PCSK9^D374Y^ and LDLR signaling pathways. To achieve this goal, the transfection of the pcDNA3+PCSK9^D374Y^-FLAG plasmid into HepG2 cells was carried out for obtaining the production of the PCSK9 mutant protein in the hepatic system. After the treatment of transfected HepG2 cells with T9 at the concentration of 100 µM, a decrease of PCSK9^D374Y^-FLAG protein level by 41.2 ± 2.6% compared to the untreated transfected HepG2 sample was achieved, in agreement with the reduction of HNF-1α protein level by 19.8 ± 8.2% (Figure 2). In addition, T9 reduces the PCSK9-WT protein levels by 10.0 ± 5.2% in untransfected treated HepG2 cells compared to untransfected control cells (Figure S1A,B), even though this reduction is not significant, suggesting that its activity is more selective for the mutant PCSK9.

In parallel, T9 induces the activation of the LDLR pathway through the increase of the LDLR protein levels due to an up-regulation of the mature SREBP-2 transcription factor (Figure 3). Our findings confirm also the T9 ability to increase the LDLR protein levels by 118.5 ± 4.4% also in untransfected treated HepG2 compared to untransfected control cells, respectively (Figure S1C,D), in agreement with the ability of T9 to improve the HepG2 capability to absorb extracellular LDL [15]. Surprisingly, T9 determines a decrease of HMGCoAR protein levels by 21.7 ± 1.1% compared to untreated transfected cells. From a functional point of view, the ability of transfected HepG2 cells to absorb the LDL from the extracellular space was impaired compared to the untransfected cells, demonstrating that the expression of mutant PCSK9^D374Y^ affects the activity of LDLR on the cell surface (Figure 4A). Our results clearly underline that the treatment with T9 renews the ability of transfected HepG2 cells to absorb extracellular LDL, suggesting a restoring of the active amount of LDLR on the membrane of hepatic cells (Figure 4A). For demonstrating this hypothesis, ICW experiments were performed without permeabilizing cells for detecting only the surface LDLR. As shown in Figure 4B, the active amount of LDLR protein level on the cell surface is reduced after transfection with PCSK9^D374Y^-FLAG plasmid compared to untransfected cells, but after treatment with T9, a complete restore of the active amount of LDLR proteins on the cell membrane was achieved (Figure 4B).

Taking into account all this information, it is possible to affirm that there is an interesting similarity between the hypocholesterolemic behaviors of T9 and P5. In fact, both peptides show the same dual-inhibitory hypocholesterolemic behavior, even though the former is selective towards the mutant PCSK9, whereas the latter is selective toward the PCSK9-WT pathway modulation, respectively. Indeed, P5 produces an increase of the LDLR protein levels through an augmentation of the SREBP-2 transcription factor activation and a decrease of PCSK9-WT protein level via a decrease of HNF-1α, leading to an enhanced capacity of HepG2 cells to clear LDL from the extracellular space [16]. On the contrary, P5 does not diminish the HMGCoAR protein levels, suggesting that T9 acts with a new hypocholesterolemic mechanism of action. Indeed, it is known that the activation of the SREBP-2 transcription factor should lead to the increase of both LDLR and HMGCoAR protein levels [22], whereas T9 not only directly inhibits the HMGCoAR activity, but also reduces its protein production at the intracellular level, suggesting a distinct hypocholesterolemic behavior.

The literature provides some evidence regarding the existence of bioactive peptides from different food sources with multifunctional activity [23]. In this dynamic field, we have discovered a new class of bioactive peptides. In particular, T9 is able to impart the in vitro hypocholesterolemic effect by modulating both PCSK9^D374Y^ and LDLR signaling pathways with a dual mechanism of action. The dual inhibition of HMGCoAR and PCSK9^D374Y^ permits to overcome the problem derived from the only HMGCoAR inhibition and the consequent cellular cholesterol depletion, which implies an increase of PCSK9 levels through the SREBP-mediated regulation. In conclusion, our results highlight the discovery of a new peptide with a complementary positive effect on the regulation of cholesterol metabolism. This approach might open the route toward a new area of food bioactive peptides with cholesterol-lowering properties. For this reason, further in vivo investigation on suitable animal models would be needed.

## Supplementary Materials

The following documents are available online at https://www.mdpi.com/2072-6643/11/7/1665/s1, Brief description of material and methods; Figure S1: Effect of T9 on the PCSK9-WT and LDLR protein level variations.

## Abbreviation

BSA: bovine serum albumin; CV, cardiovascular; DMEM, Dulbecco’s modified Eagle’s medium; FBS, fetal bovine serum; GOF, Gain-of-function; HepG2, human hepatoma G2; HMGCoAR, 3-hydroxy-3-methylglutaryl Co-Enzyme A Reductase; HNF1-α, Hepatocyte Nuclear Factor 1 alpha; HRP, horseradish peroxidase; ICW, in-cell western; LDL, low density lipoprotein; LDL-C, LDL cholesterol; LDLR, LDL receptor; PBS, phosphate buffered saline; PCSK9, Proprotein Convertase Subtilisin/Kexin 9; PMSF, phenylmethanesulfonyl fluoride; PPIs, protein-protein interactions; RT, room temperature; SDS, Sodium Dodecyl Sulphate; SREBP, Sterol Regulatory Element Binding Proteins; WT, wild type.
