# Supplementary material for: Lupin Peptide T9 (GQEQSHQDEGVIVR) Modulates the Mutant PCSK9D374Y Pathway: in vitro Characterization of its Dual Hypocholesterolemic Behavior
Source: Nutrients. 2019 Jul 20;11(7):1665. doi: 10.3390/nu11071665 (PMC6683083; doi:10.3390/nu11071665)
Supplement: Supplementary file 1 [file nutrients-11-01665-s001.pdf]

Brief description of material and methods

2. Materials and Methods

2.1. Materials

Dulbecco's modified Eagle's medium (DMEM), stable L-glutamine, fetal bovine serum (FBS), phosphate buffered saline (PBS), penicillin/streptomycin, chemiluminescent reagent, and 96-well plates were purchased from Euroclone (Milan, Italy). HMGCoAR assay kit, bovine serum albumin (BSA), RIPA buffer, the antibody against β-actin, monoclonal anti-flag antibody, Janus Green B, formaldeyde, HCl and H_2SO_4 were from Sigma-Aldrich (St. Louis, MO, USA). The antibody against HMGCoAR was bought from Abcam (Cambridge, UK). The antibody against hepatocyte nuclear factor 1α (HNF-1α) was bought from GeneTex (Irvine, CA, USA). The antibodies against SREBP-2, rabbit Ig horseradish peroxidase (HRP), mouse Ig-HRP, phenylmethanesulfonyl fluoride (PMSF), Na-orthovanadate inhibitors were purchased from Santa Cruz Biotechnology Inc. (Santa Cruz, CA, USA); the inhibitor cocktail Complete Midi from Roche (Basel, Switzerland). Mini protean TGX pre-cast gel 7.5% and Mini nitrocellulose Transfer Packs were purchased from BioRad (Hercules, CA, USA). The TMB substrate was bought from Pierce (Rockford, IL, USA). The LDL-DyLight™ 550 was from Cayman Chemical (Ann Arbor, MI, USA). The synthetic peptide T9 was synthesized by the company GeneScript (Piscataway, NJ, USA) at >95% purity. TurboFect Transfection Reagent was from Thermo Scientific (Waltham, MA, USA).

2.2. HMGCoAR Activity Assay

The assay buffer, NADPH, substrate solution, and HMGCoAR were provided in the HMGCoAR Assay Kit (Sigma). The experiments were carried out following the manufacturer's instructions and conditions, which were reported in the Supplementary materials. Briefly, peptide T9 was tested in a concentration range from 10^{-5} to 10^{-3} M or vehicle (C), at 37 °C. The absorbance at time 0 and 10 min were recorded. The HMGCoAR-dependent oxidation of NADPH and the inhibition properties of lupin peptides were measured by the absorbance reduction at 340 nm read by a microplate reader Synergy H1 from Biotek.

2.3. Cell Culture Conditions and Transfection

HepG2 cell line was bought from ATCC (HB-8065, ATCC from LGC Standards, Milan, Italy) and was cultured following the conditions previously described [4]. Detailed information is reported in the Supplementary materials. A total of 3 × 10^4 HepG2 cells/well were seeded in 96-well plates, respectively. The following day, cells at 70–90% of confluence were transfected with the mixture containing 1.0 μg pcDNA3+PCSK9^{D374Y}-FLAG plasmid and 2.0 μL TurboFect Transfection Reagent in 100 μL of serum-free DMEM for 48 h. After 24 h, transfected HepG2 cells were treated with peptide T9 (100 μM) and incubated for 24 h at 37 °C under 5% CO_2 atmosphere.

2.4. Western Blot Analysis

Transfected HepG2 cells (1.5 × 10^5 cells/well) were treated with 100 μM T9 for 24 h. After each treatment, cells were collected and lysated in 30.0 μL ice-cold lysis buffer containing: RIPA buffer + inhibitor cocktail + 1:100 PMSF + 1:100 Na-orthovanadate. After centrifugation at 16,060 g for 15 min at 4 °C, the supernatant was recovered and total proteins were quantified by the Bradford method. A total of 50.0 μg of total proteins were loaded on a pre-cast 7.5% Sodium Dodecyl Sulphate-Polyacrylamide (SDS-PAGE) gel at 130 V for 45 min. Subsequently, the proteins were transferred to a nitrocellulose membrane (Mini nitrocellulose Transfer Packs,) using a Trans-Blot Turbo at 1.3 A, 25 V for 7 min. Target proteins, on milk blocked membrane, were detected by primary antibodies as follows: rabbit anti-SREBP-2, rabbit anti-LDLR, anti-HMGCoAR, anti-HNF1-α, anti-FLAG (for detecting PCSK9^{D374Y}), and anti-β-actin. Secondary antibodies conjugated with HRP and a chemiluminescent reagent were used to visualize target proteins and their signal was quantified

using the Image Lab Software (Biorad). The internal control β-actin was used to normalize loading variations.

2.5. In Cell-Western

Transfected HepG2 cells were treated with or w/o 100 μM T9 and vehicle (H_2O) for 2 h at 37 °C under 5% CO_2 atmosphere. Treated HepG2 cells were fixed in 4% paraformaldehyde for 20 min at room temperature (RT). Cells were washed 5 times with 100 μL of PBS/well (each wash was for 5 min at RT) and the endogenous peroxides activity was quenched adding 3% H_2O_2 in PBS for 20 min at RT. Non-specific sites were blocked with 100 μL/well of 5% BSA in PBS for 1.5 h at RT. LDLR primary antibody solution (1:3000 in 5% BSA in PBS, 25 μL/well) was incubated O/N at 4 °C. Subsequently, the primary antibody solution was discarded and each sample was washed 5 times with 100 μL/well of PBS (each wash was for 5 min at RT). Goat anti-rabbit Ig-HRP secondary antibody solution (1:6000 in 5% BSA in PBS, 50 μL/well) was added and incubated 1 h at RT. The secondary antibody solution was washed 5 times with 100 μL/well of PBS (each wash for 5 min at RT). Freshly prepared TMB substrate (100 μL/well), was added and the plate was incubated at RT until the color was developed. The reaction was then stopped with 2 M H_2SO_4 and the absorbance at 450 nm was measured using the Synergy H1 fluorescent plate reader from Biotek. Cells were stained by adding 1 × Janus green stain, incubating for 5 min at RT. The dye was removed and the sample washed 5 times with water. Afterward 0.1 mL 0.5 M HCl per well were added and incubated for 10 min. After 10 s shaking, the OD at 595 nm was measured using the Synergy H1 fluorescent plate reader from Biotek.

2.6. Assay for Evaluation of Fluorescent LDL Uptake by HepG2 Cells

After transfection, a total of 3 × 10^4 HepG2 cells/well were seeded in 96-well and treated with 100 μM T9 or vehicle (H_2O) for 24 h. At the end of the treatment period, the culture medium was replaced with 75 μL/well LDL-DyLight™ 550 working solution. The cells were additionally incubated for 2 h at 37 °C and then the culture medium was aspirated and replaced with PBS (100 μL/well). The degree of LDL uptake was measured using the Synergy H1 fluorescent plate reader from Biotek (excitation and emission wavelengths 540 and 570 nm, respectively).

2.7. Statistically analysis

Statistical analyses were carried out by One-way ANOVA (Graphpad Prism 6) followed by Dunnett's test. Values were expressed as means ± SD; *P*-values < 0.05 were considered to be significant.

3. Results

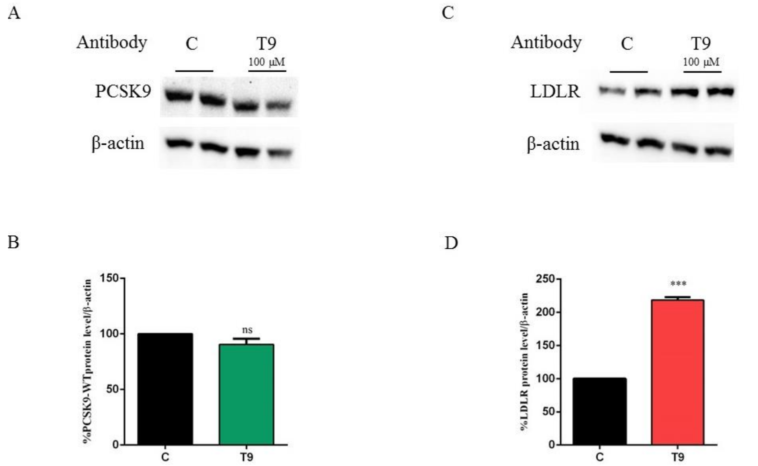

Figure S1. Effect of T9 on the PCSK9-WT and LDLR protein level variations. T9 peptide induces increases of LDLR protein level in untrasfected cells without affecting the PCSK9 intracellular pathway. (***) $p < 0.0001$
